# Endocrine-responsive lobular carcinoma of the breast: features associated with risk of late distant recurrence

**DOI:** 10.1186/s13058-019-1234-9

**Published:** 2019-12-30

**Authors:** Fabio Conforti, Laura Pala, Eleonora Pagan, Giuseppe Viale, Vincenzo Bagnardi, Giulia Peruzzotti, Tommaso De Pas, Nadia Bianco, Rossella Graffeo, Elena Guerini Rocco, Andrea Vingiani, Richard D. Gelber, Alan S. Coates, Marco Colleoni, Aron Goldhirsch

**Affiliations:** 1 Division of Medical Oncology for Melanoma, Sarcoma, and Rare Tumors, IEO, European Institute of Oncology IRCCS, Via Ripamonti 435, 20141 Milan, Italy; 2 Department of Statistics and Quantitative Methods, University of Milan-Bicocca, Milan, Italy; 3 Department of Pathology, IEO, European Institute of Oncology IRCCS & State University of Milan, Milan, Italy; 4 Division of Data Management, IEO, European Institute of Oncology IRCCS, Milan, Italy; 5 Division of Medical Senology, IEO, European Institute of Oncology IRCCS, Milan, Italy; 6 Institute of Oncology (IOSI) and Breast Unit (CSSI) of Southern Switzerland, Bellinzona, Switzerland; 7 Division of Pathology and Laboratory Medicine, IEO, European Institute of Oncology IRCCS, Milan, Italy; 8 Department of Biostatistics and Computational Biology, Dana-Farber Cancer Institute, Harvard Medical School, Harvard T.H. Chan School of Public Health, and Frontier Science & Technology Research Foundation, Boston, USA; 9 International Breast Cancer Study Group and University of Sydney, Sydney, Australia; 10 Scientific Directorate, IEO, European Institute of Oncology IRCCS, Milan, Italy; 11 MultiMedica San Giuseppe Hospital, Milan, Italy

**Keywords:** Lobular carcinoma, Late distant recurrence risk, KiCST5

## Abstract

**Background:**

Invasive lobular carcinomas (ILCs) account for 10–15% of all breast cancers. They are characterized by an elevated endocrine responsiveness and by a long lasting risk of relapse over time. Here we report for the first time an analysis of clinical and pathological features associated with the risk of late distant recurrence in ILCs.

**Patients and methods:**

We retrospectively analyzed all consecutive patients with hormone receptor–positive ILC operated at the European Institute of Oncology (EIO) between June 1994 and December 2010 and scheduled to receive at least 5 years of endocrine treatment.

The aim was to identify clinical and pathological variables that provide prognostic information in the period beginning 5 years after definitive surgery. The cumulative incidence of distant metastases (CI-DM) from 5 years after surgery was the prospectively defined primary endpoint.

**Results:**

One thousand eight hundred seventy-two patients fulfilled the inclusion criteria. The median follow-up was 8.7 years.

Increased tumor size and positive nodal status were significantly associated with higher risk of late distant recurrence, but nodal status had a significant lower prognostic value in late follow-up period (DM-HR, 3.21; 95% CI, 2.06–5.01) as compared with the first 5 years of follow-up (DM-HR, 9.55; 95% CI, 5.64–16.2; heterogeneity *p* value 0.002).

Elevated Ki-67 labeling index (LI) retained a significant and independent prognostic value even after the first 5 years from surgery (DM-HR, 1.81; 95% CI 1.19–2.75), and it also stratified the prognosis of ILC patients subgrouped according to lymph node status.

A combined score, obtained integrating the previously validated Clinical Treatment Score post 5 years (CTS5) and Ki-67 LI, had a strong association with the risk of late distant recurrence of ILCs.

**Conclusion:**

We identified factors associated with the risk of late distant recurrence in ER-positive ILCs and developed a simple prognostic score, based on data that are readily available, which warrants further validation.

## Introduction

Invasive lobular carcinoma (ILC) is the second most common histologic subtype of breast cancer (BC) and accounts for approximately 10–15% of all BCs. Compared with invasive carcinoma nonspecial type (NST; previously: invasive ductal carcinomas, IDC), it has different biology and natural history [1, 2].

Classic ILCs are typically of low or intermediate histologic grade and low to intermediate mitotic index. More than 90% of cases express estrogen (ER) and/or progesterone receptors (PgR) and rarely show HER2 protein overexpression or gene amplification [3].

In gene expression profiling studies, more than 80% of ILCs are classified as luminal A, with very few cases classified as HER2-enriched or basal-like molecular subtypes [2].

From a clinical perspective, these biological features translate in an elevated endocrine responsiveness and also in a peculiar pattern of risk of relapse, characterized by a very long lasting risk over time [3, 4].

In a large stage-matched analysis with long follow-up, it has been reported that ILC and IDC disease-free survival (DFS) and overall survival (OS) curves crossed over time, with an initial favorable prognosis for ILCs becoming unfavorable with longer follow-up in this subgroup [4].

Results of studies exploring the efficacy of extended endocrine therapy beyond 5 years showed that the risk of late recurrence could be reduced [5–10].

Recognition of the magnitude of the residual recurrence risk of ER-positive BC patients after 5 years of endocrine therapy is therefore useful, since it can help to decide whether to extend therapy [ 5–10].

Several retrospective studies assessed the correlation between the clinical and pathological features of ER-positive BCs and the risk of distant recurrence after the first 5 years of endocrine therapy [11–13].

Consistent results showed that the tumor size and number of positive nodes were the strongest factors significantly and independently associated with risk of late distant recurrence [11–13].

For both factors, the strength of association with the risk of distant recurrence was similar during both the early and late periods of follow-up [11–13].

In contrast, other factors that were of some additional prognostic relevance during the first 5 years were of less, or no, additional relevance thereafter.

Tumor grade and Ki-67 labeling index (LI) were significant independent prognostic factors during the first 5 years, but were of only small relevance thereafter [11–13].

PgR and HER2 status were independently associated with the clinical outcome only during years 0 to 5 [11–13].

None of these studies took into account the histological subtype of BC analyzed.

Since the vast majority of ER+ BC are IDCs (currently, invasive breast carcinoma NST), results obtained were dominated by this tumor subtype. Uncertainty remains on their validity for other BC subtypes, especially ILCs, whose pattern of risk of relapse over time significantly differs from that of IDCs [4].

Here we report for the first time an evaluation of the influence of clinical and pathological characteristics on late recurrence risk of women with ER-positive, early-stage ILCs, who were scheduled to receive adjuvant endocrine therapy for at least 5 years.

## Methods

We extracted information from our prospectively collected institutional database on all consecutive ILC patients operated at the European Institute of Oncology (EIO) between June 1994 and December 2010.

We included in our analysis only hormone receptor–positive ILCs (steroid hormone receptor status was classified as positive for ≥ 1% immunoreactive tumor cells).

Patients with a previous primary tumor, with a mixed lobular/ductal histotype, or with missing information on lymph nodes status, primary tumor size, ER and PgR status, and Ki-67 LI were excluded.

Histological types were classified according to the World Health Organization criteria and the Armed Forces Institute of Pathology criteria [14, 15]. Tumor grade, peritumoral vascular invasion (PVI), ER and PgR status, Ki-67 LI, and HER2 overexpression and/or amplification were evaluated as previously reported [16–21].

The original pathology reports were used.

We also extracted a sub-cohort from the hormone receptor–positive IDCs treated at EIO in the same time period. Patients in the sub-cohort were 1:1 matched to patients in the ILC cohort according to patients’ age group (< 50, 50–59, and ≥ 60 years old), nodal status, T stage, year of surgery (before 2003, 2003–2006, 2007–2010), and tumor subtype classification (luminal A-like and luminal B-like according to St. Gallen 2013 classification) [16–21].

### Statistical analysis

The primary objective of this study was to determine whether the clinical pathological features of ILCs provide prognostic information in the period beginning 5 years after definitive surgery.

The cumulative incidence of distant metastases (CI-DM) from 5 years after surgery was the prospectively defined primary endpoint.

The other endpoints evaluated were disease-free survival (DFS; measured both from the date of surgery and from 5 years after surgery), overall survival (OS; measured from the date of surgery), and CI-DM measured from the date of surgery.

Active follow-up was conducted to determine patient status as of July 2018. Surviving patients were censored at the date of the last follow-up.

Patients were followed up with physical examination every 6 months, annual mammography and breast ultrasound, blood tests every 6–12 months, and further evaluations only in case of symptoms.

When possible, the status of women not presenting at the institute for scheduled follow-up visits for more than 1 year was obtained by telephone contact.

Events considered in the DFS computations were relapse (categorized as loco-regional events, including ipsilateral breast recurrence, and distant metastases), appearance of a second primary cancer (including contralateral breast cancer), or death, whichever occurred first.

OS was defined as the time from surgery until the date of death (from any cause).

The DFS and OS functions were estimated using the Kaplan–Meier method. The log-rank test was used to assess differences between groups.

The CI-DM curve function was estimated according to methods described by Kalbfleisch and Prentice, taking into account the competing causes of recurrence. Gray’s test was used to assess cumulative incidence differences between groups [22, 23].

Univariable and multivariable Cox proportional hazard regression models and Fine and Gray’s proportional sub-distribution hazard models were used to assess the association of clinical and histopathologic characteristics of the tumor on DFS and CI-DM, respectively. Factors included in multivariable regression analyses were histological grade (G1/G2, G3), T stage (pT1/2, pT3/4), nodal status (pN0, pN1/2/3), PgR (< 20% and ≥ 20%), Ki-67 (divided using institutional median value as < 20%, ≥ 20%), and HER2 overexpression (negative, positive) [17].

The heterogeneity of grade, tumor stage, nodal status, PgR, Ki-67 LI, and HER2 effects on the risk of DM within each considered time period (≤ 5 years versus > 5 years) was assessed by including interaction terms between the factor of interest and time period in the regression models.

We also assessed the association between the Clinical Treatment Score post 5 years (CTS5) and CI-DM after the first 5 years of follow-up.

CTS5 is a prognostic tool to estimate risk of late distant recurrence that was developed and validated on the TransATAC and BIG1-98 data set [24, 25].

The CTS5 model includes information on age (continuous, in years), tumor size (continuous, in cm), quadratic tumor size, nodal status (five groups: 0, negative; 1, one positive; 2, two to three positive; 3, four to nine positive; and 4, nine positive), and grade (three groups: 1, low; 2, intermediate; and 3, high) and is given by:

CTS5 = 0.438 × nodes + 0.988 × (0.093 × tumor size − 0.001 × (tumor size)^2^ + 0.375 × grade + 0.017 × age) [24, 25].

The added prognostic values, beyond that obtained from CTS5, of other factors such as the HER2 status, the expression of ER, PgR, or Ki-67, was evaluated using likelihood ratio test (LRT), comparing a regression model with only CTS5 as covariate with a model including CTS5 and the factor of interest [24, 25].

All analyses were performed with SAS software v. 9.4 (SAS Institute, Cary, NC). All tests were two-sided, and *p* values < 0.05 were considered statistically significant.

### Treatment received

All patients received breast conserving surgery or total mastectomy, plus axillary sentinel lymph node biopsy or complete axillary dissection [20].

Systemic adjuvant therapy was recommended according to the contemporary St. Gallen treatment guidelines [17, 20, 26–28].

We included patients that were scheduled to receive endocrine therapy for at least 5 years, regardless of actual adherence.

Adjuvant endocrine therapy in pre-menopausal patients included tamoxifen alone for 5 years or the combination of tamoxifen for 5 years plus a luteinizing hormone releasing hormone analog for a minimum of 2 years [20, 26]. In post-menopausal patients, an aromatase inhibitor commonly formed part of endocrine therapy either as only endocrine therapy for 5 years or after 2–3 years of tamoxifen [17, 20, 26]. Post-menopausal patients at low risk or with comorbidities received tamoxifen alone.

Details on adjuvant endocrine therapies are reported in Table 1.

**Table 1:** Distribution of patient baseline characteristics

	Number	Percentage
Age group		
< 40	75	4.0
40–49	570	30.4
50–59	540	28.8
60+	687	36.7
Median age (IQR)	54 (47–64)	
Menopausal status		
Pre-menopausal	788	42.1
Post-menopausal	1084	57.9
pN		
pN0	1103	58.9
pN1	449	24.0
pN2	131	7.0
pN3	189	10.1
pT		
pT1	1060	56.6
pT2	612	32.7
pT3/4	200	10.7
Tumor grade		
G1	279	14.9
G2	1232	65.8
G3	209	11.2
Unknown	152	8.1
Vessel invasion		
No	1797	96.0
Yes	75	4.0
Local treatment		
Mastectomy w/o RT	355	19.0
Mastectomy w RT	245	13.1
Quadrantectomy w/o RT	36	1.9
Quadrantectomy w RT	1236	66.0
Adjuvant treatment		
ET	1481	79.1
CT+ET	391	20.9
Hormonal therapy		
Pre-menopausal		
TAM	107	13.6
TAM+LHRH	556	70.5
AI+LHRH	58	7.4
Other	67	8.5
Post-menopausal		
TAM	483	44.6
AI	463	42.7
Other	138	12.7
Receptor status		
Incompletely expressed (ER < 50 or PgR < 50)	841	44.9
Highly expressed (ER ≥ 50 and PgR ≥ 50)	1031	55.1
Ki-67		
< 20%	1375	73.5
≥ 20%	497	26.5
HER2		
Not expressed	1727	92.3
Intense and complete	72	3.8
Unknown	73	3.9
ER mean (SD)/median (IQR)	83 (17)/90 (80–95)	
PgR mean(SD)/median (IQR)	51 (37)/60 (10–90)	
Ki-67 mean(SD)/median (IQR)	15 (9)/14 (9–20)	

After 2005, patients with node-positive or node-negative tumors with worse prognostic features may have been treated with extended endocrine therapy for further 5 years of treatment with tamoxifen if they were pre-menopausal patients or AI if they were post-menopausal [29].

In patients at higher risk and/or with features of uncertain endocrine responsiveness, chemotherapy was added [17, 20, 26]. Anthracycline-containing chemotherapy [i.e., doxorubicin and cyclophoshamide (AC), for four courses] was considered as the first option in patients with higher risk disease; in case of comorbidities or patients’ preferences, classical CMF (oral cyclophosphamide, methotrexate, and fluorouracil) for three to six courses was considered [30, 31].

## Results

### Clinical and pathological characteristics associated with higher risk of late recurrence in ILCs

Between June 1994 and December 2010, 1872 patients with hormone receptor–positive ILC operated at the EIO fulfilled the inclusion criteria for the analysis.

The baseline characteristics of patients are shown in Table 1. The median follow-up time (FUP) was 8.7 years, for a total of 13,883 person-years (PY).

We observed 520 DFS events of which 205 were distant metastases. Of these, 279 DFS events, including 116 distant metastases, occurred within the first 5 years and 241 DFS events, including 89 distant metastases, occurred beyond 5 years after surgery (Additional file 1: Table S1).

In the first 5 years of follow-up, the DFS and DM yearly rates were 3.4% (95% CI, 3.0–3.8) and 1.4% (95% CI, 1.2–1.7), respectively.

In the period beyond the first 5 years after surgery, the DFS and DM yearly rates were 4.3% (95% CI, 3.8–4.9) and 1.6% (95% CI, 1.3–1.9), respectively.

We first assessed the prognostic value of clinical and pathological tumor features in predicting CI-DM and DFS, separately in the first 5 years after surgery and beyond the first 5 years of FUP.

Univariable analyses showed that in the first 5 years of follow-up, all variables analyzed had significant prognostic value for DM (Table 2).

**Table 2:** Prognostic factors of early (≤ 5 years) and late (> 5 years) distant recurrences in ILCs, univariable and multivariable analysis

	≤ 5 years					> 5 years								Heterogeneity *p* value (univariate analyses)
	Univariable analysis					Univariable analysis					Multivariable analysis for late DM (> 5 years)			
	*N*	Distant events/PY	HR	95% CI	*p* value	*N*	Distant events/PY	HR	95% CI	*p* value	HR	95% CI	*p* value	
Ki-67 (%)														
< 20%	1375	70/6110	Ref.			1060	52/4046	Ref.			Ref.			
≥ 20%	497	46/2165	1.85	1.27–2.68	0.001	366	37/1562	1.81	1.19–2.75	0.005	1.90	1.17–3.07	0.009	0.93
HER2^a^														
Not expressed	1727	102/7629	Ref.			1318	77/5009	Ref.			Ref.			
Intense and complete	72	10/300	2.38	1.26–4.52	0.008	45	3/159	1.13	0.35–3.62	0.84	1.18	0.34–4.06	0.79	0.43
pN														
pN0	1103	16/5017	Ref.			901	29/3397	Ref.			Ref.			
pN1/2/3	769	100/3259	9.55	5.64–16.2	< 0.001	525	60/2212	3.21	2.06–5.01	< 0.001	2.70	1.68–4.34	< 0.001	0.002
Grade^b^														
G1/2	1511	64/6767	Ref.			1190	71/4649	Ref.			Ref.			
G3	209	27/871	3.24	2.07–5.07	< 0.001	135	8/568	0.89	0.44–1.83	0.76	0.55	0.26–1.16	0.12	0.003
pT														
pT1/2	1672	85/7447	Ref.			1298	68/5140	Ref.			Ref.			
pT3/4	200	31/828	3.24	2.15–4.90	< 0.001	128	21/468	3.31	2.05–5.33	< 0.001	2.42	1.42–4.12	0.001	0.91
PgR														
< 20%	555	57/2392	Ref.			402	26/1727	Ref.			Ref.			
≥ 20%	1317	59/5883	0.42	0.29–0.61	< 0.001	1024	63/3881	1.10	0.69–1.74	0.69	1.03	0.64–1.67	0.90	0.001

^a^For patients with HER2, unknown 4 events occurred within the first 5 years of FUP and 9 beyond 5 years

^b^For patients with grade, unknown 25 events occurred within the first 5 years of FUP and 10 beyond 5 years

In the period beyond the first 5 years, factors significantly associated with the risk of DM were positive nodal status (HR, 3.21; 95% CI, 2.06–5.01), T3/4 stage (HR, 3.31; 95% CI, 2.05–5.33), and high Ki-67 LI (HR, 1.81; 95% CI, 1.19–2.75; Table 2).

The strength of association with the risk of DM was not significantly different in the first 5 years and in the subsequent period of follow-up for Ki-67 LI and T stage (Table 2).

Positive nodal status had a significant lower prognostic value in the late follow-up period (HR, 3.21; 95% CI, 2.06–5.01) as compared with the first 5 years of FUP (HR, 9.55; 95% CI, 5.64–16.2; heterogeneity *p* value 0.002; Table 2).

Analysis exploring the association between clinico-pathological variables and DFS in the first 5 years after surgery and beyond the first 5 years of FUP showed similar results (Additional file 1: Table S2).

We further focused our analyses on prognostic factors associated with risk of late recurrence in ILCs, as no data are available in literature on this topic.

Among all patients, 1426 women had at least 5 years of FUP and remained disease-free in the first 5 years after surgery.

In multivariable analysis, factors retaining significant and independent prognostic value for risk of late DM were nodal status, T stage, and Ki-67 LI (Table 2).

A sensitivity analysis was conducted excluding 45 HER2-positive tumors and 63 HER2 unknown tumors obtaining similar results (data not shown).

Similar results were obtained also in multivariable analyses for DFS (Additional file 1: Table S2).

**Fig. 1:**
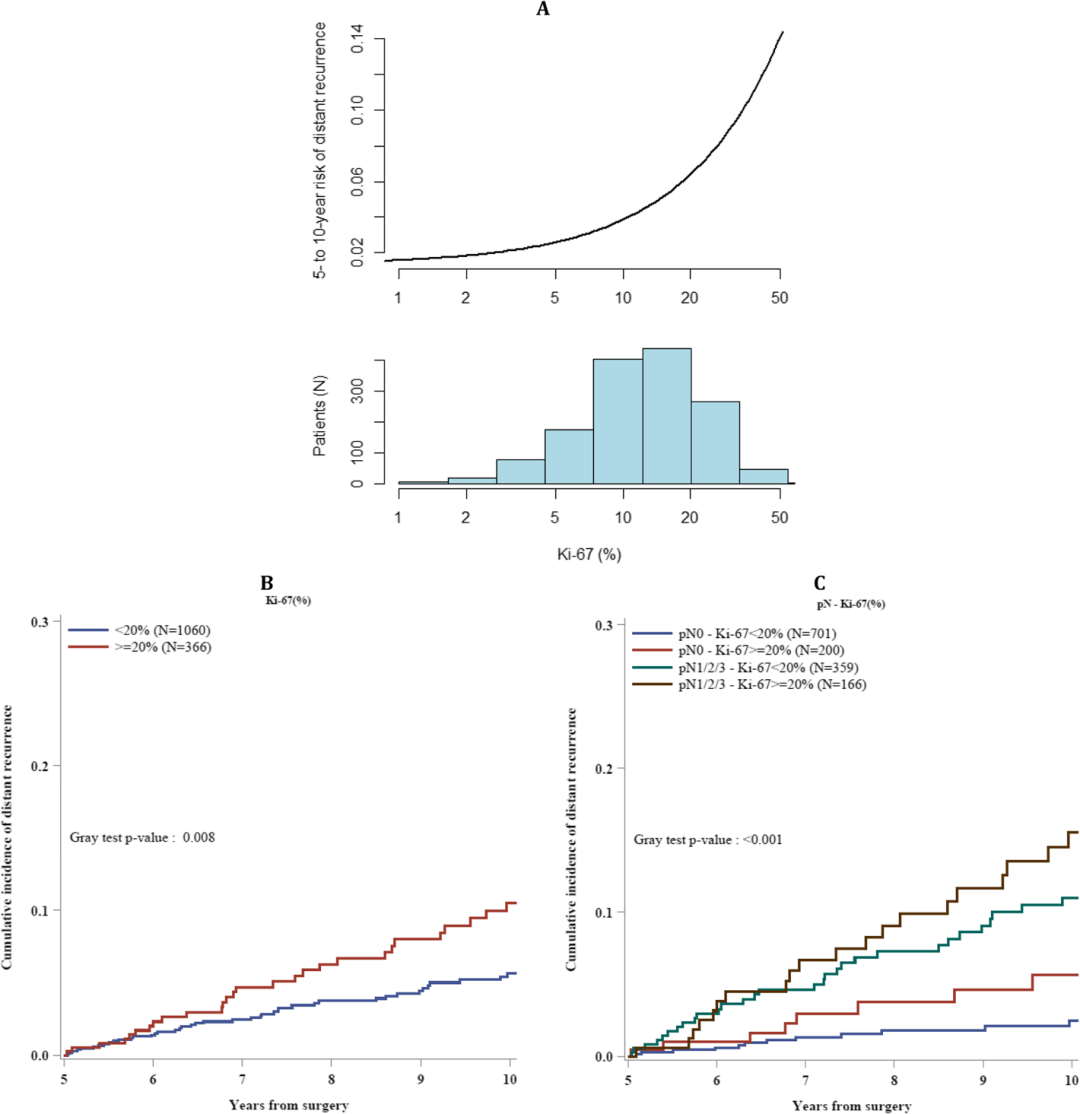
Cumulative incidence of distant recurrences after the first 5 years from surgery in ILCs, according to Ki-67 Index as continuum after log-transformation (**a**); according to Ki-67 index categorized as < 20% or ≥ 20% (**b**); and according to nodal status and Ki-67 index categorized as < 20% or ≥ 20% (**c**)

Figure 1a shows the relationship between Ki-67 LI (log transformed) and risk of DM between years 5 and 10, together with a representation of the frequency distribution of Ki-67 LI in the group of ILCs analyzed.

It is evident that there is a steady rising of the risk of DM with increasing values of Ki-67 LI.

Ki-67 LI, categorized as below or equal and above 20%, stratified ILC patients in two groups with significantly different risk of late distant recurrence (Gray test *p* value 0.008; HR, 1.81; 95% CI 1.19–2.75; Fig. 1b).

The absolute risk of DM in years 5 to 10 of FUP was 5.6% (95% CI, 4.1–7.5) in the Ki-67 < 20% group and 10.5% (95% CI, 7.1–14.6) in the Ki-67 ≥ 20% group (Fig. 1b).

Ki-67 also stratified the prognosis of ILC patients subgrouped according to lymph node status (pN0 and pN1/2/3; Fig. 1c).

In lymph node–negative ILCs, tumors with Ki-67 ≥ 20% had a risk of late DM almost three times higher than those with Ki-67 < 20% (HR, 2.88; 95% CI, 1.29–6.45; Table 3).

**Table 3:** Prognostic factors of late (> 5 years) distant recurrences in ILCs by lymph node status

	pN0 (*N* = 901)					pN1/2/3 (*N* = 525)					*p* value for interaction with pN
	Multivariable analysis					Multivariable analysis					
	*N*	Events/PY	HR	95% CI	*p* value	*N*	Events/PY	HR	95% CI	*p* value	
Ki-67 (%)											
< 20%	701	15/2584	Ref.			359	37/1462	Ref.			
≥ 20%	200	14/812	2.88	1.29–6.45	0.01	166	23/750	1.52	0.85–2.72	0.16	0.06
HER2^a^											
Not expressed	846	24/3116	Ref.			472	53/1893	Ref.			
Intense and complete	26	2/85	3.48	0.80–15.1	0.10	19	1/74	0.52	0.07–3.85	*0.52*	0.21
Grade^b^											
G1/2	791	23/2993	Ref.			399	48/1657	Ref.			
G3	72	3/279	0.63	0.18–2.18	0.46	63	5/289	0.52	0.21–1.29	0.16	0.19
pT											
pT1/2	864	24/3272	Ref.			434	44/1868	Ref.			
pT3/4	37	5/125	3.26	1.24–8.57	0.02	91	16/344	2.04	1.15–3.64	0.02	0.09
PgR											
< 20%	245	5/1005	Ref.			157	21/722	Ref.			
≥ 20%	656	24/2391	1.81	0.69–4.77	0.23	368	39/1490	0.75	0.43–1.32	0.32	0.14

^a^Twenty-nine patients pN0 with missing information (3 events/195 PY), 34 patients pN1/2/3 with missing information (6 events/244 PY)

^b^Thirty-eight patients pN0 with missing information (3 events/124 PY), 63 patients pN1/2/3 with missing information (7 events/266 PY)

In lymph node–positive tumors, the risk of late DM was 50% higher in tumors with Ki-67 ≥ 20% (HR, 1.52; 95% CI, 0.85–2.72; Table 3).

In the group of lymph node–negative tumors with Ki-67 < 20% (701 of 1426 ILCs), there was a very low incidence of late DM (absolute distant recurrence risk in years 5 to 10, 2.5%; 95% CI, 1.3–4.3; Fig. 1c).

Lymph node–positive tumors with Ki-67 ≥ 20% displayed the highest incidence of late DM (absolute distant recurrence risk in years 5 to 10, 15.5%; 95% CI, 9.8–22.5; Fig. 1c).

Similarly, Ki-67 LI stratified DFS of ILC patients subgrouped by lymph node status (Additional file 1: Table S3).

These data show that in ILCs, KI-67 LI retained a significant and unchanged prognostic value for risk of DM in the late period of FUP as compared with the first 5 years after surgery, whereas positive nodal status had a significantly reduced prognostic value in late follow-up.

Finally, we performed a sensitivity analysis in which we analyzed separately patients that received or not adjuvant chemotherapy and we confirmed similar results in both groups (data not shown).

### Features associated with late distant recurrence risk are partially different in ILCs and IDCs

To confirm that these findings are specific for ILCs, we analyzed a cohort of patients with IDCs, 1:1 matched to patients in the ILC cohort according to patients’ age group, nodal status, T stage, year of surgery, and tumor subtype classification (luminal A-like and luminal B-like according to St. Gallen 2013 classification).

The flowchart for the patient’s selection and baseline characteristics of the matched cohorts of ductal and lobular cancers are shown respectively in Additional file 1: Figure S1 and Table S4.

We found that in IDCs, the strength of association between nodal status and the risk of DM was not significantly different in the first 5 years and in the subsequent period of follow-up (HR in first 5 years, 5.80; 95% CI, 3.70–9.08; HR after 5 years, 7.13; 95% CI, 3.69–13.8; *p* heterogeneity 0.61; Additional file 1: Table S5).

Ki-67 LI was significantly associated with risk of DM only in the first 5 years of follow-up (HR, 2.73; 95% CI, 1.89–3.94; Additional file 1: Table S5) and lost its prognostic value in the subsequent period of FUP (HR, 1.57; 95% CI, 0.91–2.70; *p* heterogeneity 0.10; Additional file 1: Table S5).

### KI-67 LI provided significant independent prognostic information when added to the CTS5 in ILCs

The Clinical Treatment Score post 5 years (CTS5) is built on nodal status, tumor size, grade, and patient age, and it has been demonstrated that it is significantly associated with late DM risk in ER+BCs.

In populations affected in the vast majority of cases by IDCs, CTS5 score was able to identify three groups of patients with respectively low risk of late distant metastases (i.e., late risk of DM < 5% if CTS5 was < 3.13), intermediate (i.e., DM risk between 5 and 10% when CTS5 ranged between 3.13 and 3.86), and high risk (DM risk > 10% when CTS5 > 3.86) [24, 25].

We therefore investigated whether CTS5 was also associated with late DM risk of ILCs. For this analysis, we excluded patients with missing data needed to calculate CTS5, leading to a sample size of 1301 women.

**Fig. 2:**
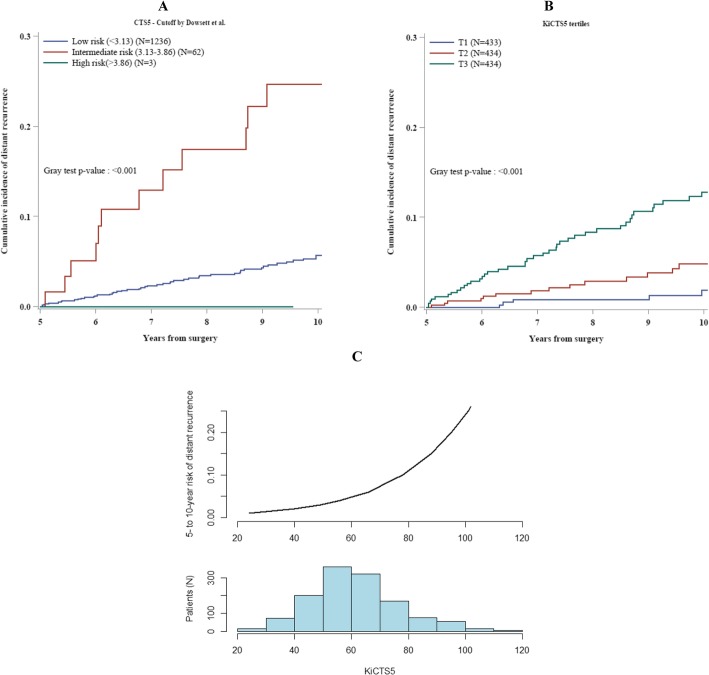
Cumulative incidence of distant recurrences after the first 5 years from surgery in ILCs, according to CTS5-predicted groups of risk—low risk (CTS5 < 3.13 and predicted DM risk < 5%), intermediate (3.13 < CTS5 < 3.86 and predicted DM risk between 5 and 10%), and high risk (CTS5 > 3.86 and predicted DM risk > 10%)—(**a**); according to KiCTS_5_ tertiles (**b**); and according to KiCTS_5_ as continuum (**c**)

Figure 2a shows the actual risk of late DM in patients with ILCs categorized in the three groups of risk predicted using the CTS5 cut-offs identified and validated in the original paper [24, 25].

Patients in both low and intermediate CTS5 groups had an actual DM risk higher than predicted: the absolute risk of DM in years 5 to 10 was respectively 5.7% (95% CI 4.2–7.5%) in the low CTS5 group and 24.7% (95% CI 13.4–37.8%) in the intermediate CTS5 group (Fig. 2a).

Only 3 patients were classified in the CTS5 high-risk group, but they did not have any recurrence.

We assessed whether other clinical and pathological variables could improve the prognostic value of the CTS5 score in ILCs.

The ER and PgR levels and HER2 status did not provide significant prognostic value for late DM when added to CTS5 assessed as continuous variable (Additional file 1: Table S6).

The only variable that provided significant independent prognostic information when added to the CTS5 was the Ki-67 LI after logarithmic transformation (*p* value LRT 0.04; Additional file 1: Table S6).

Combining the CTS5 and ln(Ki-67) values, we obtained a final combined score that we named KiCTS_5_ (i.e., *Ki*-67 index and *C*linical *T*reatment *S*core post *5* years).

The relationship between KiCTS_5_ and risk of DM between years 5 and 10 is shown in Fig. 2c, together with a representation of the frequency distribution of KiCTS_5_ values in the group of ILCs analyzed. It is evident that there is a steady rising of the risk of distant recurrence with increasing values of KiCTS_5_.

ILC patients categorized in three groups on the basis of KiCTS_5_ tertiles—low, intermediate, and high KiCTS_5_—had significantly different risk of late distant recurrence (Gray test *p* value < 0.001; Fig. 2b).

The group of patients with low KiCTS_5_ had a 1.9% (95% CI, 0.7–4.2) absolute risk of DM in years 5 to 10 of follow-up (Fig. 2b). Patients with intermediate KiCTS_5_ had an absolute risk of DM of 4.8% (95% CI, 2.7–7.9) while patients with high KiCTS_5_ had an absolute risk of 12.8% (95% CI, 9.3–16.8) (Fig. 2b).

## Discussion

The risk of BC recurrence varies considerably over time, being strongly influenced by clinical and pathological variables. The main factor that influences the pattern of BC recurrence over time is its hormone receptor status [11, 32, 33].

Patients with ER-positive tumors continue to have a higher risk of relapse, including distant metastases, during years 5 to 25 [11, 33].

Studies performed to identify factors associated with higher risk of recurrence after 5 years of endocrine therapy included mainly IDCs, and uncertainty remains on their validity for other rare BC subtypes [ 11–13, 24, 34–37].

Here we report for the first time an analysis of the association between clinico-pathological factors and the risk of late DM, performed specifically in ILCs. Similar to previous studies, mainly conducted in IDCs, we found that nodal status and T stage retained a significant prognostic value beyond the first 5 years of follow-up.

A relevant difference, however, is that in ILCs, positive nodal status had an impressively lower prognostic value in late follow-up period as compared with the first 5 years after surgery.

On the contrary, the strength of association between Ki-67 LI and risk of DM did not significantly change over time and Ki-67 LI was able to stratify the prognosis of patients with both node-negative and node-positive disease.

Since our data show that in ILCs, nodal status alone is not useful to accurately predict the risk of late recurrence, we therefore combined several clinico-pathological factors to obtain a score with stronger prognostic value for late recurrence. Using a statistical parsimoniuous approach that allows to avoid overfitting, we obtained a combined score that integrates the previously validated CTS5 score with Ki-67 LI [38]. This score was able to stratify the prognosis of ILC patients in the late follow-up period and to identify a large group of patients with ILC who have a very low risk of late DM (650 of 1301 patients, with a cumulative distant recurrence risk of 2.6% during years 5 to 10).

The reasons why tumors in more advanced stage and with higher Ki-67 LI at diagnosis retain a higher risk of relapse after 5 years of endocrine treatment are unknown. A higher tumor burden at diagnosis could be associated with a higher amount of biological tumor heterogeneity [39]. Higher tumor Ki-67 could reflect deeper alterations of the mechanisms that regulate the cell cycle [ 40, 41].

It could be speculated that both these conditions are responsible for a lower degree of tumor endocrine responsiveness, leading to an incomplete eradication of micrometastases that restart growth when treatments are stopped. In this regard, it has been demonstrated that alteration of the mechanisms that control cell cycle, and in particular of the cyclin D/cyclin-dependent kinases 4 and 6 (CDK4/6)/retinoblastoma (Rb) pathway, is one of the most relevant mechanisms of endocrine resistance in metastatic BCs, with some data obtained from neoadjuvant treatments [42–44].

A strength of our study is that it has been performed in a single institution. All included patients had pathological evaluation carried out by the same team of pathologists, ensuring consistent pathological reporting. We had a very large cohort of ILCs with a considerable number of late distant recurrences.

Yet our study has several limitations.

Similar to other studies of this kind so far published, the recurrence rates reported here are in women who were scheduled to receive at least 5 years of endocrine therapy, not in those who actually completed treatment, because detailed data on treatment adherence were not available.

Furthermore, after 2005, data on the efficacy of extended adjuvant endocrine (EET) treatment in nodal positive, ER+ BC have become available [29]. After this period, some patients treated in our institute with ER+ and node-positive tumors or tumors with node-negative but worse prognostic features started to receive extended endocrine treatment (tumors diagnosed and treated 5 years earlier). The percentage of recurrences observed in our data are therefore derived from a cohort of patients that includes also a subgroup treated for more than 5 years. The number of patients that received EET included in our analysis is limited, but we are not able to precisely quantify it: this represents a weakness shared by almost all studies to date performed in such field.

However, it seems unlikely that such weaknesses would have a substantial effect on the generalizability of our findings regarding the association between the variables analyzed and the risk of late recurrences in ILCs.

Obviously, further validation of the prognostic value of KiCST5 score in an independent cohort of patients with ILCs is needed, especially to evaluate whether and how much the reported interlaboratory variability in the assessment of Ki67-LI could limit the generalizability of the prognostic value of the KiCST5.

## Conclusion

The main finding of our analysis is that ILCs display specific characteristics in terms of relationship between clinical and pathological features and risk of late recurrence.

Future research that focused on the evaluation of factors affecting BC late recurrence risk should take into account BC histological subtypes as a relevant variable.

From a clinical point of view, given the significant loss of prognostic value of nodal status for late recurrence risk in ILCs, a broader assessment including other clinico-pathological features, and in particular Ki-67 index, is probably needed to better estimate the late DM risk of patients, that is of great importance for selecting patients suitable or not for extended adjuvant endocrine therapy (EET).

Indeed, data available from RCTs showed that EET significantly reduced the risk of late DM in patients’ populations with endocrine responsive BCs, but the absolute amount of such reduction is limited on average [5–10].

Since there are no definitive predictive biomarkers useful to identify patients that derive benefit from EET, the choice to administer or not EET relies on a cost-benefit assessment done case by case and that largely depends on the estimate of the patients’ residual risk of DM after the first 5 years of endocrine treatment [45].

In particular, patients with low risk of late DM could be those to whom properly propose to avoid EET [45].

## Supplementary information

**Additional file 1: Table S1.** reports Type and distribution of events over time. **Table S2.** reports Prognostic factors of early (≤5 years) and late (> 5 years) DFS events in ILCs, univariate and multivariate analysis. **Table S3.** reports Prognostic factors of late (> 5 years) DFS in ILCs by lymph-node status. **Table S4.** reports Distribution of patient baseline characteristics according to intrinsic and histological subtype – matched groups. **Table S5.** reports Prognostic factors of early (≤ 5 years) and late (> 5 years) distant recurrences in IDCs and ILCs, univariable analysis. **Table S6.** reports the Likelihood ratio test *p*-value of the addition of HER2, ER, PgR and ln(Ki-67) variables to CTS5 considered as continuous variable. **Figure S1.** reports Flowchart for patient’s selection and matching

## Data Availability

Yes
