# Supplementary material for: Endocrine-responsive lobular carcinoma of the breast: features associated with risk of late distant recurrence
Source: Breast Cancer Res. 2019 Dec 30;21:153. doi: 10.1186/s13058-019-1234-9 (PMC6937973; doi:10.1186/s13058-019-1234-9)
Supplement: Supplementary file 1 — Additional file 1: Table S1. reports Type and distribution of events over time. Table S2. reports Prognostic factors of early (≤5 years) and late (> 5 years) DFS events in ILCs, univariate and multivariate analysis. Table S3. reports Prognostic factors of late (> 5 years) DFS in ILCs by lymph-node status. Table S4. reports Distribution of patient baseline characteristics according to intrinsic and histological subtype – matched groups. Table S5. reports Prognostic factors of early (≤ 5 years) and late (> 5 years) distant recurrences in IDCs and ILCs, univariable analysis. Table S6. reports the Likelihood ratio test p-value of the addition of HER2, ER, PgR and ln(Ki-67) variables to CTS5 considered as continuous variable. Figure S1. reports Flowchart for patient’s selection and matching [file 13058_2019_1234_MOESM1_ESM.pdf]

Table S1. Type and distribution of events over time.

	N
**All FUP years**	
No event	1352
Loco-regional events	160
Distant metastases	205
Other	155
Alive	1689
Dead	183
**First five years from surgery**	
DFS events	279
Yearly rate, × 100 PY (95% CI)	3.4 (3.0-3.8)
DM events	116
Yearly rate, × 100 PY (95% CI)	1.4 (1.2-1.7)
**Beyond five year after surgery**	
DFS events	241
Yearly rate, × 100 PY (95% CI)	4.3 (3.8-4.9)
DM events	89
Yearly rate, × 100 PY (95% CI)	1.6 (1.3-1.9)

Table S2. Prognostic factors of **early** (≤5 years) and **late** (>5 years) **DFS** events in **ILCs**, univariate and multivariate analysis.

	≤5 years					>5 years								*Heterogeneity p-value (univariate analyses)*
	**Univariable analysis**					**Univariable analysis**					**Multivariable analysis for late DFS (>5 years)**			
	**N**	**Distant events/PY**	**HR**	**95% CI**	***P-value***	**N**	**Distant events/PY**	**HR**	**95% CI**	***P-value***	**HR**	**95% CI**	**P-value**	
**Ki-67 (%)**														
<20%	1375	184/6110	Ref.			1060	149/4046	Ref.			Ref.			
≥20%	497	95/2165	1.46	1.14-1.87	*0.003*	366	92/1562	1.56	1.20-2.03	*<0.001*	1.71	1.26-2.32	*<0.001*	*0.73*
**HER2** [a]														
Not expressed	1727	247/7629	Ref.			1318	206/5009	Ref.			Ref.			
Intense and complete	72	23/300	2.40	1.56-3.67	*<0.001*	45	13/159	1.99	1.13-3.48	*0.02*	1.79	0.99-3.19	*0.05*	*0.67*
**pN**														
pN0	1103	99/5017	Ref.			901	126/3397	Ref.			Ref.			
pN1/2/3	769	180/3259	2.83	2.21-3.62	*<0.001*	525	115/2212	1.38	1.07-1.77	*0.01*	1.20	0.92-1.57	*0.17*	*<0.001*
**Grade** [b]														
G1/2	1511	186/6767	Ref.			1190	187/4649	Ref.			Ref.			
G3	209	55/871	2.32	1.72-3.14	*<0.001*	135	31/568	1.33	0.91-1.94	*0.15*	0.93	0.61-1.42	*0.74*	*0.04*
**pT**														
pT1/2	1672	224/7447	Ref.			1298	206/5140	Ref.			Ref.			
pT3/4	200	55/828	2.24	1.67-3.00	*<0.001*	128	35/468	1.90	1.32-2.71	*<0.001*	1.75	1.19-2.57	*0.004*	*0.49*
**PgR**														
<20%	555	105/2392	Ref.			402	83/1727	Ref.			Ref.			
≥20%	1317	174/5883	0.67	0.53-0.85	*0.001*	1024	158/3881	0.86	0.66-1.13	*0.28*	0.85	0.64-1.11	*0.23*	*0.16*

[a] For patients with HER2 unknown 9 events occurred within the first 5 years of FUP and 22 beyond 5 years.
[b] For patients with grade unknown 38 events occurred within the first 5 years of FUP and 23 beyond 5 years.

Table S3. Prognostic factors of **late** (>5 years) **DFS** in **ILCs** by **lymph-node status.**

	pN0 (N=901)					pN1/2/3 (N=525)					*P-value for interaction with pN*
	Multivariable analysis					Multivariable analysis					
	**N**	**Events/PY**	**HR**	**95% CI**	***P-value***	**N**	**Events/PY**	**HR**	**95% CI**	***P-value***	
**Ki-67 (%)**											
<20%	701	82/2584	Ref.			359	67/1462	Ref.			
≥20%	200	44/812	1.61	1.04-2.50	*0.03*	166	48/750	1.84	1.20-2.82	*0.005*	*0.61*
**HER2** [a]											
Not expressed	846	107/3116	Ref.			472	99/1893	Ref.			
Intense and complete	26	7/85	2.29	1.05-4.98	*0.04*	19	6/74	1.49	0.63-3.53	*0.37*	*0.12*
**Grade** [b]											
G1/2	791	102/2993	Ref.			399	85/1657	Ref.			
G3	72	16/279	1.13	0.63-2.06	*0.68*	63	15/289	0.77	0.43-1.39	*0.38*	*0.17*
**pT**											
pT1/2	864	118/3272	Ref.			434	88/1868	Ref.			
pT3/4	37	8/125	1.61	0.77-3.37	*0.21*	91	27/344	1.81	1.15-2.84	*0.01*	*0.88*
**PgR**											
<20%	245	43/1005	Ref.			157	40/722	Ref.			
≥20%	656	83/2391	0.79	0.54-1.15	*0.21*	368	75/1490	0.83	0.55-1.25	*0.37*	*0.50*

[a] 29 patients pN0 with missing information (12 events/195 PY), 34 patients pN1/2/3 with missing information (10 events/244 PY).
[b] 38 patients pN0 with missing information (8 events/124 PY), 63 patients pN1/2/3 with missing information (15 events/266 PY).

Figure S1. Flowchart for patient's selection and matching.

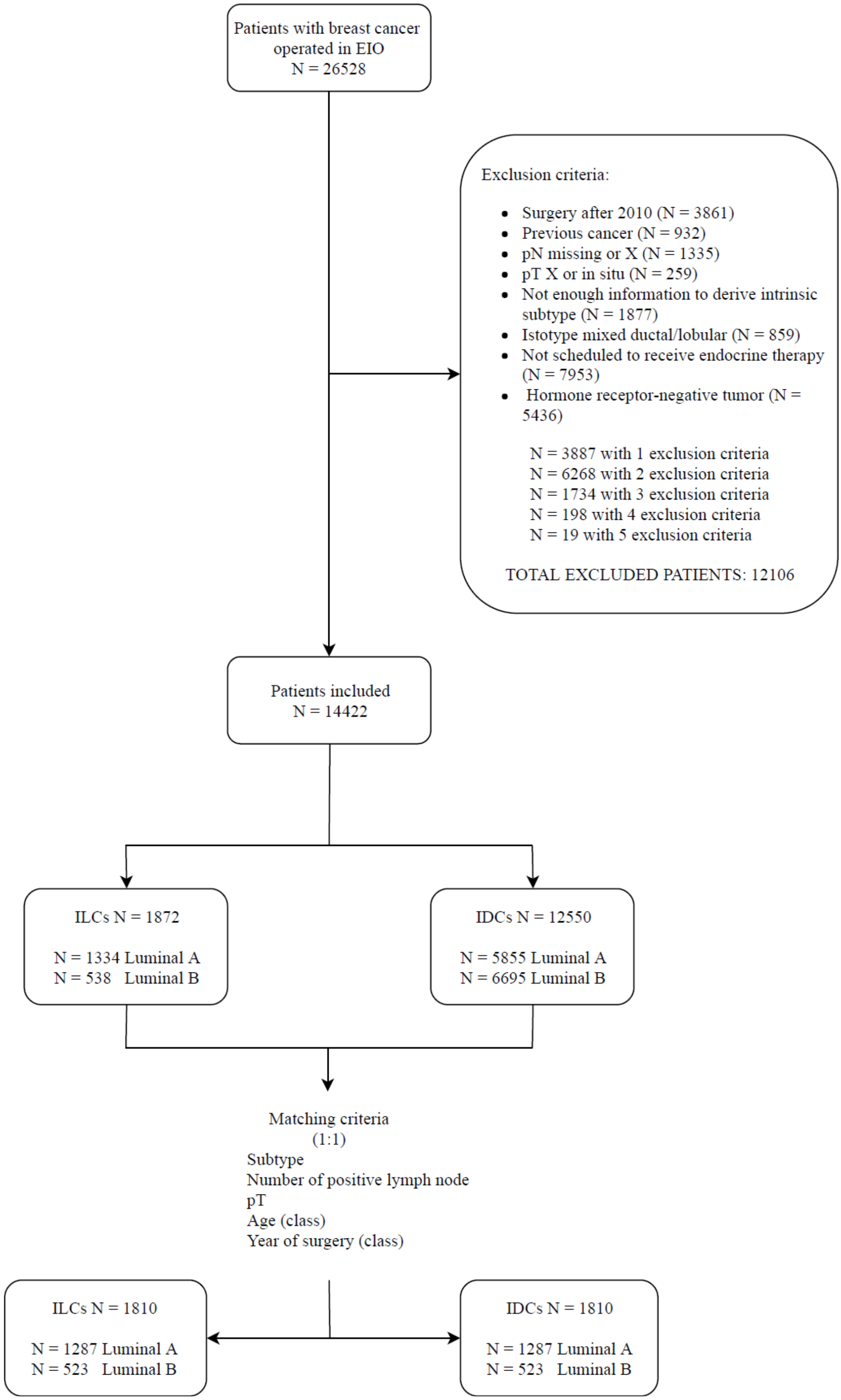

Table S4. Distribution of patient baseline characteristics according to intrinsic and histological subtype – matched groups

	Lobular (N=1810)		Ductal (N=1810)		
	N	%	N	%	*P-value*
**Matching variables**					
**Subtype**					
Luminal A	1287	71	1287	71	
Luminal B	523	29	523	29	
**pN**					
pN0	1076	60	1076	60	
pN1	439	24	439	24	
pN2	129	7	129	7	
pN3	166	9	166	9	
**pT**					
pT1	1055	58	1055	58	
pT2	608	34	608	34	
pT3/4	147	8	147	8	
**Age group**					
<50	638	35	638	35	
50-59	519	29	519	29	
60+	653	36	653	36	
**Year of surgery**					
Before 2003	520	29	520	29	
2003-2006	641	35	641	35	
2007-2010	649	36	649	36	
**Other variables**					
**Menopausal status**					*0.31*
Premenopausal	777	43	747	41	
Postmenopausal	1033	57	1063	59	
**Tumor grade**[a]					*<0.001*
G1	276	15	423	23	
G2	1201	66	923	51	
G3	196	11	275	15	
Unknown	137	8	189	11	
**Vessel invasion**					*<0.001*
No	1739	96	1281	71	
Yes	71	4	529	29	
**Local treatment**					*<0.001*
Mastectomy w/o RT	322	18	242	14	
Mastectomy w RT	230	13	217	12	
Quadrantectomy w/o RT	35	2	25	1	
Quadrantectomy w RT	1223	67	1326	73	
**Adjuvant treatment**					*0.01*
ET	1440	80	1379	76	
CT+ET	370	20	431	24	
**Receptor status**					*0.33*
Incompletely expressed (ER<50 or PgR<50)	807	45	778	43	
Highly expressed (ER≥50 and PgR≥50)	1003	55	1032	57	
**Ki-67**					*0.35*
<20%	1327	73	1302	72	
≥20%	483	27	508	28	
**ER Mean (SD) / Median (IQR)**	83 (17) / 90 (80-95)		86 (15) / 90 (85-95)		*<0.001*
**PgR Mean (SD) / Median (IQR)**	51 (37) / 60 (10-90)		53 (37) / 60 (10-90)		*0.01*
**Ki67 Mean (SD) / Median (IQR)**	15 (9) / 14 (9-20)		17 (11) / 15 (10-21)		*<0.001*

[a] Missing values are not considered in the p-value calculation

Table S5. Prognostic factors of **early** (≤ 5 years) and **late** (> 5 years) **distant recurrences** in **IDCs** and **ILCs,** univariable analysis.

	**Hystological subtype**	**≤ 5 years**					**> 5 years**					*Heterogeneit p-value*
		**N**	**Distant events/PY**	**HR**	**95% CI**	***P-value***	**N**	**Distant events/PY**	**HR**	**95% CI**	***P-value***	
**-67 (%)**												
< 20%	Lobular	1327	61/5913	Ref.			1035	50/3958	Ref.			
≥ 20%	Lobular	483	43/2111	1.96	1.33-2.90	*<0.001*	358	35/1535	1.79	1.17-2.75	*0.008*	*0.73*
< 20%	Ductal	1302	56/5700	Ref.			921	35/3228	Ref.			
≥ 20%	Ductal	508	58/2098	2.73	1.89-3.94	*<0.001*	314	21/1249	1.57	0.91-2.70	*0.11*	*0.10*
terogeneity alue						*0.23*					*0.70*	
**R2[£]**												
Not expressed	Lobular	1671	91/7399	Ref.			1288	75/4908	Ref.			
ntense and complete	Lobular	69	10/291	2.68	1.40-5.09	*0.003*	44	3/155	1.19	0.37-3.80	*0.77*	*0.23*
Not expressed	Ductal	1691	99/7322	Ref.			1164	45/4120	Ref.			
ntense and complete	Ductal	69	7/275	1.88	0.87-4.04	*0.11*	39	5/149	3.18	1.27-7.99	*0.01*	*0.42*
terogeneity alue						*0.49*					*0.19*	
pN0	Lobular	1076	14/4900	Ref.			883	26/3336	Ref.			
pN1/2/3	Lobular	734	90/3123	10.0	5.70-17.6	*<0.001*	510	59/2157	3.58	2.26-5.67	*<0.001*	*0.005*
pN0	Ductal	1076	24/4767	Ref.			778	11/2840	Ref.			
pN1/2/3	Ductal	734	90/3031	5.80	3.70-9.08	*<0.001*	457	45/1637	7.13	3.69-13.8	*<0.001*	*0.61*
terogeneity alue						*0.14*					*0.09*	
**ade[$]**												
G1/2	Lobular	1477	56/6629	Ref.			1173	68/4590	Ref.			
G3	Lobular	196	24/824	3.40	2.11-5.49	*<0.001*	130	8/550	0.96	0.47-1.97	*0.91*	*0.004*
G1/2	Ductal	1346	37/5897	Ref.			959	28/3479	Ref.			
G3	Ductal	275	42/1132	5.77	3.71-8.97	*<0.001*	159	14/615	2.77	1.43-5.33	*0.002*	*0.08*
terogeneity alue						*0.11*					*0.03*	
pT1/2	Lobular	1663	82/7414	Ref.			1295	67/5131	Ref.			
pT3/4	Lobular	147	22/609	3.21	1.99-5.17	*<0.001*	98	18/363	3.77	2.28-6.26	*<0.001*	*0.62*
pT1/2	Ductal	1663	83/7213	Ref.			1157	45/4201	Ref.			
pT3/4	Ductal	147	31/584	4.61	3.05-6.98	*<0.001*	78	11/276	3.65	1.89-7.03	*<0.001*	*0.52*
terogeneity alue						*0.26*					*0.94*	
**R**												
< 20%	Lobular	534	50/2324	Ref.			396	26/1703	Ref.			
≥ 20%	Lobular	1276	54/5700	0.44	0.30-0.65	*<0.001*	997	59/3791	1.03	0.65-1.63	*0.91*	*0.005*
< 20%	Ductal	509	49/2187	Ref.			350	28/1322	Ref.			
≥ 20%	Ductal	1301	65/5611	0.52	0.36-0.75	*<0.001*	885	28/3155	0.41	0.24-0.69	*<0.001*	*0.45*
terogeneity alue						*0.54*					*0.01*	

[£] For patients with HER2 unknown 11 events (3 ILCs, 8 IDCs) occurred within the first 5 years of FUP and 13 beyond 5 years (7 ILCs, 6 IDCs).
[$] For patients with grade unknown 59 events (24 ILCs, 35 IDCs) occurred within the first 5 years of FUP and 23 beyond 5 years (9 ILCs, 14 IDCs).

Table S6.

	-2 log L	*Likelihood-ratio test P-value*
CTS5	980.675	
CTS5 + HER2	980.670	*0.94*
CTS5 + ER	980.541	*0.71*
CTS5 + PgR	980.611	*0.80*
**CTS5 + ln(Ki-67)***	**976.784**	***0.04***

*ln: logarithmic transformation
